# Long intergenic noncoding RNA for IGF2BP2 stability suppresses gastric cancer cell apoptosis by inhibiting the maturation of microRNA-34a

**DOI:** 10.1515/med-2024-0992

**Published:** 2024-09-27

**Authors:** Yao Wang, Zhigang Guo, Zhifeng Yang, Qingyan Deng, Yueming Huang, Yanhong Chen

**Affiliations:** Department of Gastrointestinal Surgery, Zhongshan City People’s Hospital, Zhongshan, Guangdong, 528400, P.R. China; Department of Hospital Infection Management, Zhongshan City People’s Hospital, No. 2 Sunwen East Road, Shiqi District, Zhongshan, Guangdong, 528400, P.R. China

**Keywords:** gastric cancer, LINRIS, mature miR-34a, miR-34a precursor, apoptosis

## Abstract

The oncogenic role of long intergenic noncoding RNA for IGF2BP2 stability (LINRIS) has been reported in colorectal cancer. This research aimed to study its potential involvement in gastric cancer (GC). In this study, paired GC and non-tumor tissues were obtained from 64 GC patients, and the levels of LINRIS, mature microRNA-34a (miR-34a), and miR-34a precursor in these tissues were measured with RT-qPCR. Linear regression was used to analyze their correlations. The role of LINRIS overexpression and siRNA silencing in regulating the maturation of miR-34a was analyzed by RT-qPCR. Cell apoptosis was studied with flow cytometry. It was observed that LINRIS was overexpressed in GC and showed a negative correlation with mature miR-34a, but not miR-34a precursor. In GC cells, LINRIS siRNA silencing upregulated mature miR-34a level, but not miR-34a precursor level. LINRIS overexpression downregulated miR-34a level. Cell apoptosis analysis showed that LINRIS siRNA silencing and miR-34a overexpression promoted GC cell apoptosis and suppressed cell migration and invasion, while LINRIS overexpression suppressed cell apoptosis and enhanced cell migration and invasion. In addition, the effect of LINRIS overexpression was reversed by miR-34a overexpression. Therefore, LINRIS siRNA silencing in GC may promote cell apoptosis by promoting miR-34a maturation.

## 1 Introduction

Gastric cancer (GC) is a common solid and malignant tumor responsible for 5.6% of new cancer cases and 7.7% of deaths in 2020 [1,2]. It is estimated that the overall 5-year survival rate is about 70% for non-metastatic GC cases and 30% for metastatic GC cases [3,4]. However, only fewer than 20% of GC patients are diagnosed at early stages, and the prognosis is generally poor for patients with advanced disease [5]. Therefore, novel diagnostic and treatment approaches are needed.

*Helicobacter pylori* infections, pernicious anemia, poor diet, and smoking habit contribute to gastric carcinogenesis [6,7]. Besides that, gastric carcinogenesis also requires the involvement of molecular factors, and these molecular players are proven to be promising targets for cancer therapy [8,9]. For example, targeted therapy can be applied to affect gene expression, thereby regulating cancers [10]. Non-coding RNAs, including long non-coding RNAs (lncRNAs) and microRNAs (miRNAs), encode no proteins but indirectly affect protein accumulation to affect cancers [11,12]. Therefore, miRNAs and lncRNAs may be targeted to suppress cancer. However, the role of most non-coding RNAs in cancer biology is unknown. Long intergenic noncoding RNA for IGF2BP2 stability (LINRIS) is known to promote colorectal cancer (CRC) [13]. In addition, LINRIS is overexpressed in non-small cell lung cancer (NSCLC), and LINRIS siRNA silencing inhibits miRNA-10a maturation to suppress cancer cell proliferation [14]. However, its role in GC is unknown. Our preliminary deep sequencing analysis revealed the altered LINRIS expression in GC and its inverse correlation with microRNA-34a (miR-34a), which is a tumor suppressor in many cancers, including GC [15]. In addition, we also predicted that LINRIS could bind to premature miR-34a. Therefore, LINRIS and miR-34a might interact with each other to participate in GC. This research aimed to study the crosstalk between LINRIS and miR-34a in GC.

## 2 Materials and methods

### 2.1 Tissue collections

A total of 64 GC patients (40 males and 24 females, all cases were adenocarcinomas) who were admitted to Zhongshan City People’s Hospital from March 2018 to January 2020 were enrolled in this study. Their age was ranged from 41 to 68 years with an average of 54.8 years. Patients were excluded if they had recurrent GC and were treated previously. To eliminate the possible effects of other clinical disorders on gene expression, patients complicated with other clinical disorders were also excluded from this study. The 64 patients were divided into AJCC stage I/II (*n* = 30) and III/IV (*n* = 34). Prior to therapy, GC and paired non-tumor tissues were obtained using the fine needle aspiration method, confirmed by histopathological tests, and used for RNA extraction.

### 2.2 Gastric cells and transfections

Two gastric adenocarcinoma cell lines, SNU-1 and AGS, from ATCC (USA) were used and cultured at 37°C in RPMI 1640 media (Cat # 11875093, Thermo Fisher) supplemented with 10% FBS (Cat # F2442-50ML, Sigma-Aldrich) in an incubator with 5% CO_2_ and 95% humidity.

LINRIS expression vector was constructed with pcDNA3.1 (Cat # V79020, Thermo Fisher) as the backbone (Invitrogen). LINRIS siRNA, negative control (NC) siRNA, miR-34a mimic, and NC miRNA were designed and synthesized by Invitrogen. AGS and SNU-1 cells were counted and transfected with expression vector (1 μg), siRNA (50 nM), or miRNA (50 nM) per 10^8^ cells using Lipofectamine 2000 (Cat # 11668027, Thermo Fisher). The same amount of empty expression vector, NC siRNA, or NC miRNA was also transfected to serve as NC. Cells were cultured in fresh media for 48 h prior to the subsequent experiments.

### 2.3 RNA preparations

Total RNAs were extracted from GC and non-tumor tissues and AGS and SNU-1 cells using Ribozol reagent (Cat # 15596026, Thermo Fisher) and treated with DNase I (Cat # 89836, Thermo Fisher) for 2 h at 37°C to completely remove the genomic DNAs.

### 2.4 RT-qPCR

cDNA samples were prepared using SS-IV-RT system through reverse transcription. To reverse transcribe miRNAs, poly-A tail addition was performed prior to reverse transcription, which was performed using T100™ Thermal Cycler (Bio-Rad). LINRIS and miR-34a (mature and precursor) expression levels were determined by qPCR using qPCR Master Mix with SYBR Green™ (Cat # M-915-500, GoldBio) with 18S as the internal control. MiR-34a precursor level was determined using sequence-specific primers. Mature miR-34a level was determined using a sequence-specific forward primer and poly (T) reverse primer after the addition of poly(A). Ct values were processed with 2^−ΔΔCt^ method [16]. qPCR was performed using CFX384 Touch Real-Time PCR Detection System (Bio-Rad). Primer sequences were 5′-ACTCTGCCTTTGGCTTTT-3′ (forward) and 5′-ACTTTCACTCTTCCCTATGCT-3′ (reverse) for LINRIS; 5′-GGCCAGCTGTGAGTGTTTC-3′ (forward) and 5′-GGGCCCCACAACGTGCAGC-3′ (reverse) for precursor miR-34a; 5′-CTTCGGCAGCACATATACTAA-3′ (forward) and 5′-GTGCGTGTCATCCTTGCG-3′ (reverse) for precursor U6; 5′-GGCCCTGTAATTGGAATGAGTC-3′ (forward) and 5′-CCAAGATCCAACTACGAGCTT-3′ (reverse) for 18S rRNA; and 5′-TGGCAGTGTCTTAGCTGGT-3′ (forward) and oligo d(T) for mature miR-34a.

### 2.5 *In situ* hybridization (ISH)

Paraffin-embedded tissue samples were prepared using tissue sections and rehydrated in a series of graded alcohols (100, 85, 50 and 30%). After that, the sections were incubated with 3% H_2_O_2_ for 30 min and probed using digoxin-labeled probes.

### 2.6 RNA–RNA pull-down assay

*In vitro* transcripts of LINRIS, antisense of precursor miR-34a (premiR-34a), and NC RNAs were prepared using T7 reverse transcriptase and labeled with biotin at 3′ end using Pierce™ RNA 3′ End Biotinylation Kit (Cat # 20160, GoldBio). The labeled RNAs were named Bio-LINRIS, Bio-premiR-34a (anti), and Bio-NC, respectively. Bio-LINRIS, Bio-premiR-34a (anti), and Bio-NC were transfected into both AGS and SNU-1 cells. At 48 h after transfection, cells were harvested, lysed, and incubated with magnetic beads. The bounded RNAs were eluted and subjected to RT-qPCR using CFX384 Touch Real-Time PCR Detection System (Bio-Rad) to detect precursor miR-34a. Three biological replicates were included.

### 2.7 Dual luciferase activity assay

The binding site of precursor miR-34a on LINRIS was cloned into pGL2 Luciferase Reporter Vector. The vector was named LINRIS (Luci). LINRIS (Luci) was then co-transfected with precursor miR-34a (LINRIS (Luci) + PremiR-34a group) or NC miRNA (LINRIS (Luci) + NC) into cells. Luciferase activity was determined 48 h later using Firefly Luciferase Assay Kit 2.0 (Cat #30085-T, Biotium) as previously reported. The transfection of LINRIS (Luci) alone served as a control. Three biological replicates were included.

### 2.8 Cell apoptosis assay

AGS and SNU-1 cells with transfections were subjected to cell apoptosis assay. In brief, cells were cultured in a six-well cell culture plate (50,000 cells in 2 mL serum-free medium per well) at 37°C for 48 h. After washing with ice-cold PBS, cells were stained with FITC-labeled Annexin-V and PI (Cat # APOAF-20TST, Sigma-Aldrich) for 20 min in the dark and subjected to flow cytometry to separate apoptotic cells. Flow cytometry was performed using ZE5 Cell Analyzer (Cat# 12004279, Bio-Rad) and data were analyzed using FCS Express Flow Cytometry Software (version 7, De Novo Software). Cell apoptotic rate = percentage of PI-positive and Annexin-negative (Q4) cells + PI negative and Annexin positive (Q2) cells. Three biological replicates were included.

### 2.9 Transwell assay

Transwell Assay was performed using Corning^®^ Transwell^®^ and Netwell™ inserts (Cat # 003412, Corning). In brief, cells (6,000) were transferred to the upper chamber containing non-serum media, while media containing 20% FBS was added to the lower chamber to induce cell movement. Cells were cultured under the above conditions for 24 h, and the membranes were cleaned and stained to analyze the cells under a light microscope. Invading and migrating cells were counted using Image J software. Control (C) group was set to value “100.” All other groups were normalized to this group. Three biological replicates were included.

### 2.10 Nuclear fractionation assay

The nuclear and cytoplasm of AGS cells were prepared using the Nuclear/Cytosol Fractionation Kit (Cat K266, BioVision). After that, RNA isolation and RT were performed, followed by semi-quantitative PCR with GAPDH as the internal control to determine LINRIS expression. PCR was conducted using T100™ Thermal Cycler (Bio-Rad).

### 2.11 Statistical analysis

Differences among multiple transfection groups were compared by ANOVA Tukey’s test. A *p* < 0.05 was deemed statistically significant.


**Ethical approval:** The authors are accountable for all aspects of the work in ensuring that questions related to the accuracy or integrity of any part of the work are appropriately investigated and resolved. For human experiments, the trial was conducted in accordance with the Declaration of Helsinki (as revised in 2013). The study was approved by the Human Ethics Committee of Zhongshan City People’s Hospital.
**Informed consent:** Informed consent was taken from all individual participants.

## 3 Results

### 3.1 LINRIS, mature miR-34a, and miR-34a precursor levels were altered in GC

**Figure 1:**
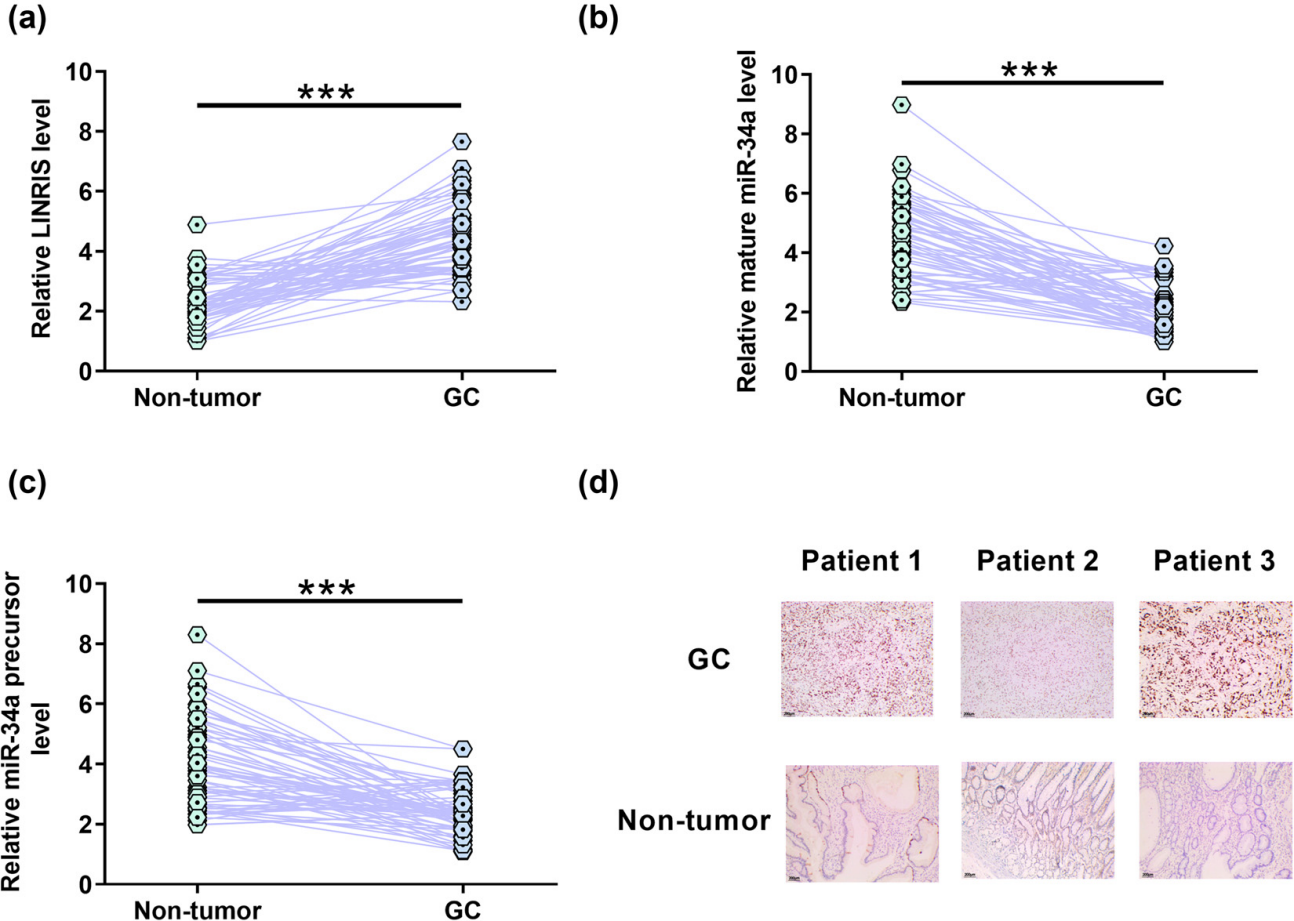
LINRIS, mature miR-34a, and miR-34a precursor levels were altered in GC. GC and paired non-tumor tissues were collected from 64 GC patients, and LINRIS (a), mature miR-34a (b), and miR-34a precursor (c) levels in these tissues were determined by RT-qPCR. ISH was performed to detect LINRIS in paired tissue samples from three GC patients. (d) ***p < 0.001.

LINRIS level and miR-34a maturation in GC were analyzed to explore their potential involvement in GC. GC tissues exhibited significantly higher LINRIS level (Figure 1a, *p* < 0.001) and significantly lower mature miR-34a (Figure 1b, *p* < 0.001) and miR-34a precursor levels (Figure 1c, *p* < 0.001). Therefore, altered LINRIS, mature miR-34a, and miR-34a precursor levels may participate in GC. LINRIS levels in paired tissue samples from three GC patients were also examined using ISH. The results showed that LINRIS signals were much stronger in GC tissues than in non-tumor tissues in two out of three cases (Figure 1d). Therefore, increased LINRIS accumulation and decreased miR-34a accumulation might participate in GC.

### 3.2 LINRIS was inversely correlated with mature miR-34a across GC tissues

**Figure 2:**
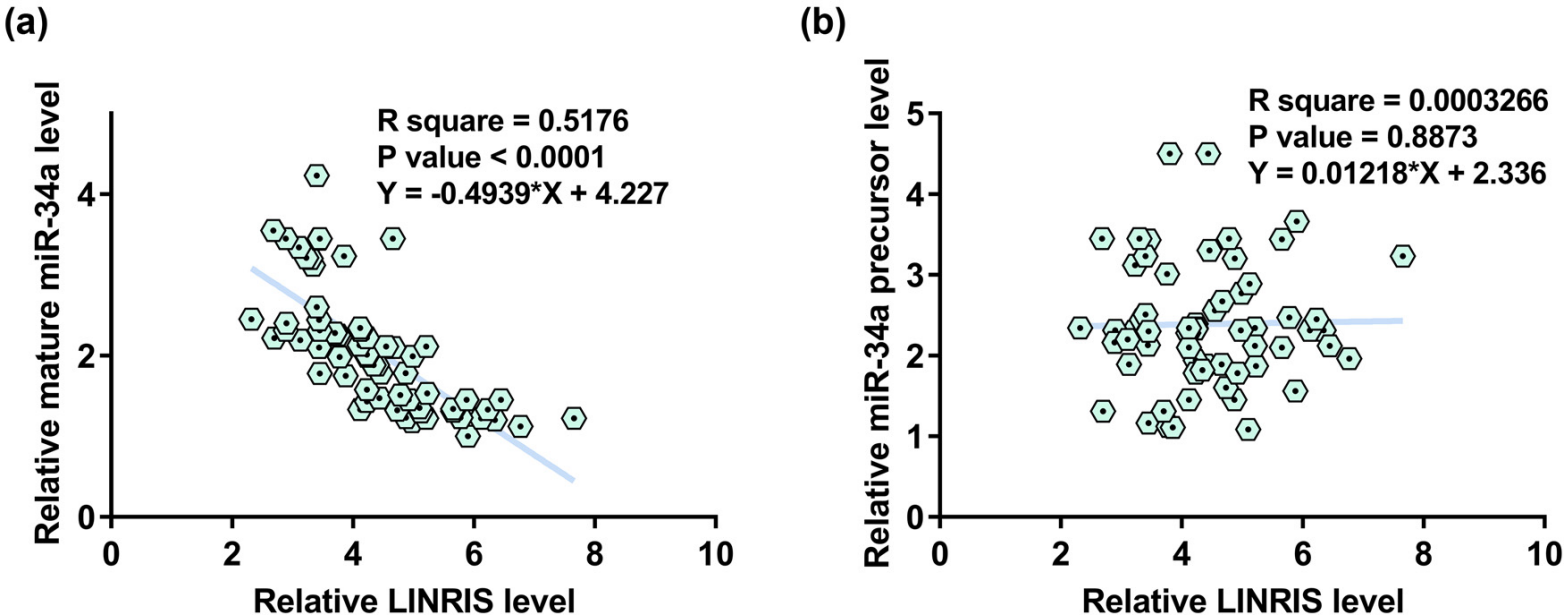
LINRIS was inversely correlated with mature miR-34a across GC tissues. The correlations between LINRIS and mature miR-34a (a) or miR-34a precursor (b) across GC tissue samples were studied with Pearson’s correlation coefficient.

Correlations suggest interaction. To this end, the correlations between LINRIS and mature miR-34a (Figure 2a) or miR-34a precursor (Figure 2b) were analyzed. Mature miR-34a and LINRIS were inversely and significantly correlated across GC tissues. In contrast, LINRIS and miR-34a precursor were not closely correlated. Therefore, it is reasonable to hypothesize that LINRIS might regulate mature miR-34a production.

### 3.3 LINRIS negatively regulated miR-34a maturation in GC cells

**Figure 3:**
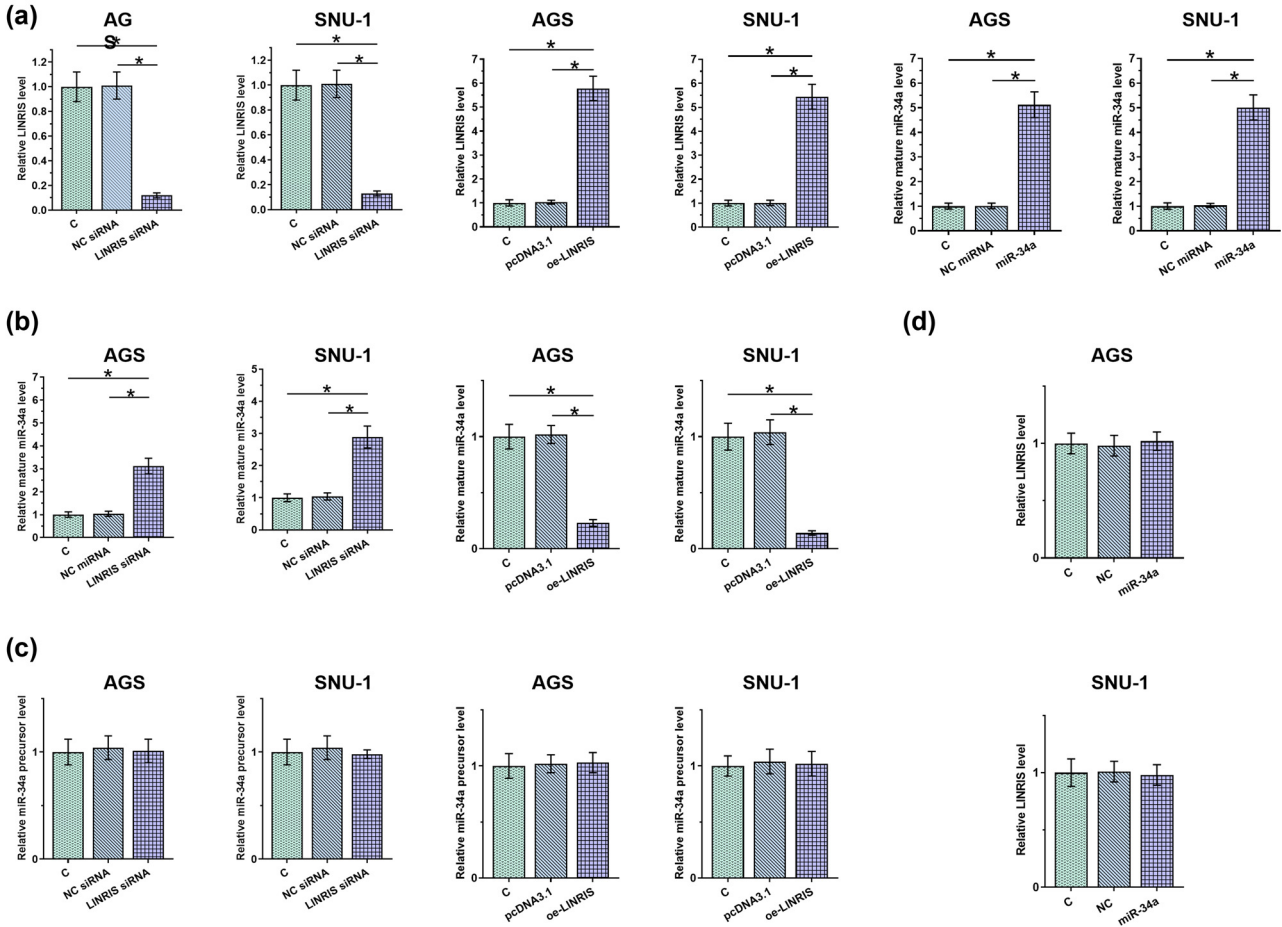
LINRIS negatively regulated miR-34a maturation in GC cells. AGS and SNU-1 cells were transfected with LINRIS expression vector, LINRIS siRNA, or miR-34a to explore their relationship. Transfections were confirmed by RT-qPCR at 48 h post-transfection (a). The effects of LINRIS siRNA silencing and overexpression on miR-34a (b) and on miR-34a precursor (c) were analyzed by RT-qPCR. The effects of miR-34a overexpression on LINRIS (d) were also analyzed by RT-qPCR. *p < 0.05.

To study the potential role of LINRIS in regulating miR-34a maturation, AGS and SNU-1 cells were transfected with LINRIS siRNA, LINRIS expression vector, or miR-34a, and the transfection was confirmed by RT-qPCR (Figure 3a, *p* < 0.05). MiR-34a level was upregulated by LINRIS siRNA silencing and downregulated by LINRIS overexpression (Figure 3b, *p* < 0.05). In contrast, miR-34a precursor level was not affected by both LINRIS overexpression and silencing (Figure 3c). Moreover, miR-34a overexpression failed to significantly affect LINRIS level (Figure 3d). Therefore, LINRIS might negatively regulate miR-34a maturation in GC cells.

### 3.4 LINRIS overexpression suppressed GC cell progression through miR-34a

**Figure 4:**
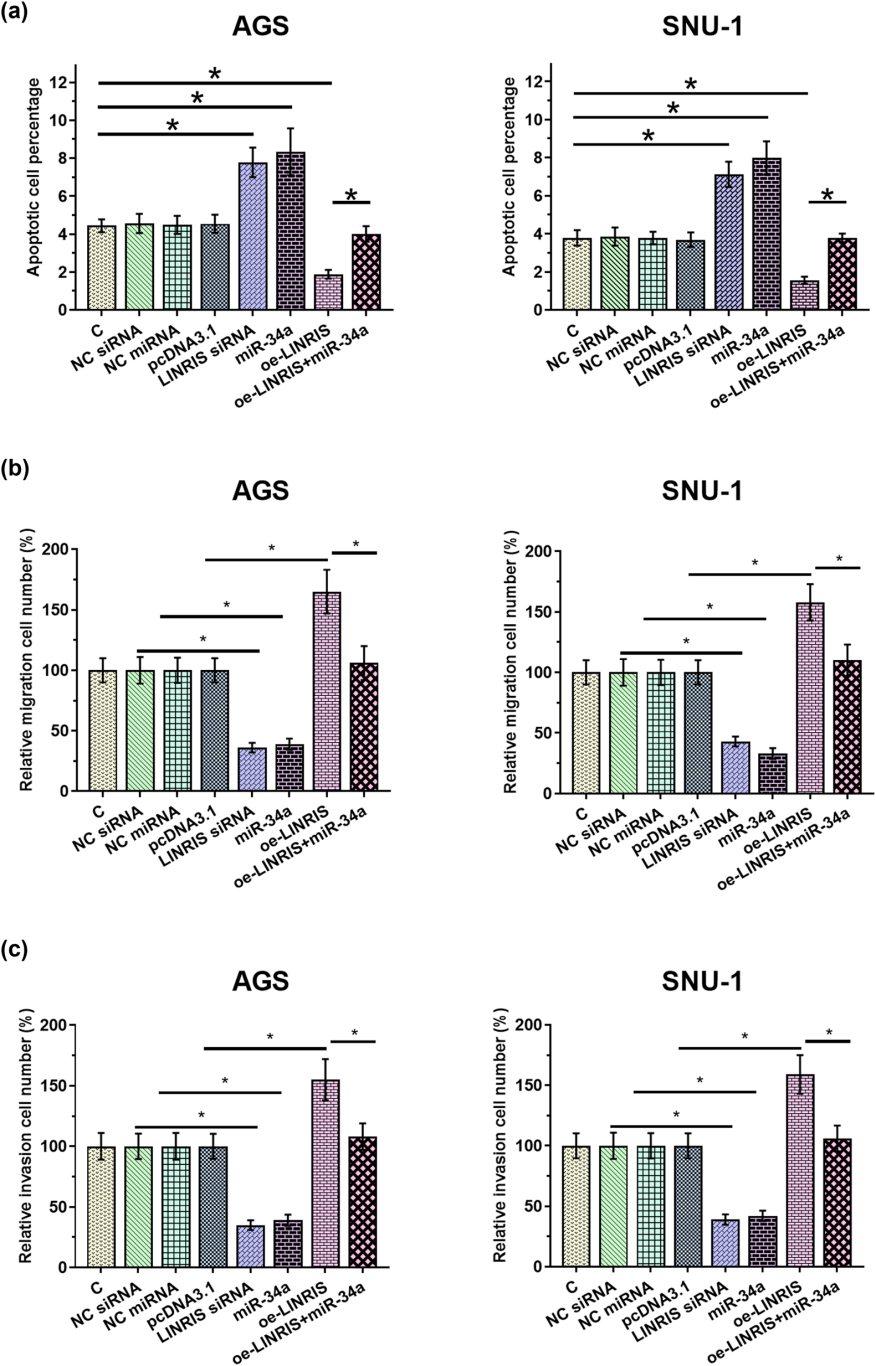
LINRIS overexpression suppressed GC cell progression through miR-34a. The roles of LINRIS overexpression, LINRIS siRNA silencing, and miR-34a overexpression in regulating GC cell apoptosis, migration, and invasion were analyzed by cell apoptosis assay and Transwell assay (a)–(c). *p < 0.05.

Cell apoptosis determines tumor progression. To this end, the roles of LINRIS overexpression, LINRIS siRNA silencing, and miR-34a overexpression in regulating GC cell apoptosis were analyzed. LINRIS siRNA silencing and miR-34a overexpression promoted GC cell apoptosis and inhibited GC cell movement, while LINRIS overexpression plays the opposite role. In addition, the effect of LINRIS overexpression was reversed by miR-34a overexpression (Figure 4a–c, *p* < 0.05). Therefore, LINRIS might suppress GC cell apoptosis through miR-34a.

### 3.5 LINRIS could sponge precursor miR-34a in GC cells

**Figure 5:**
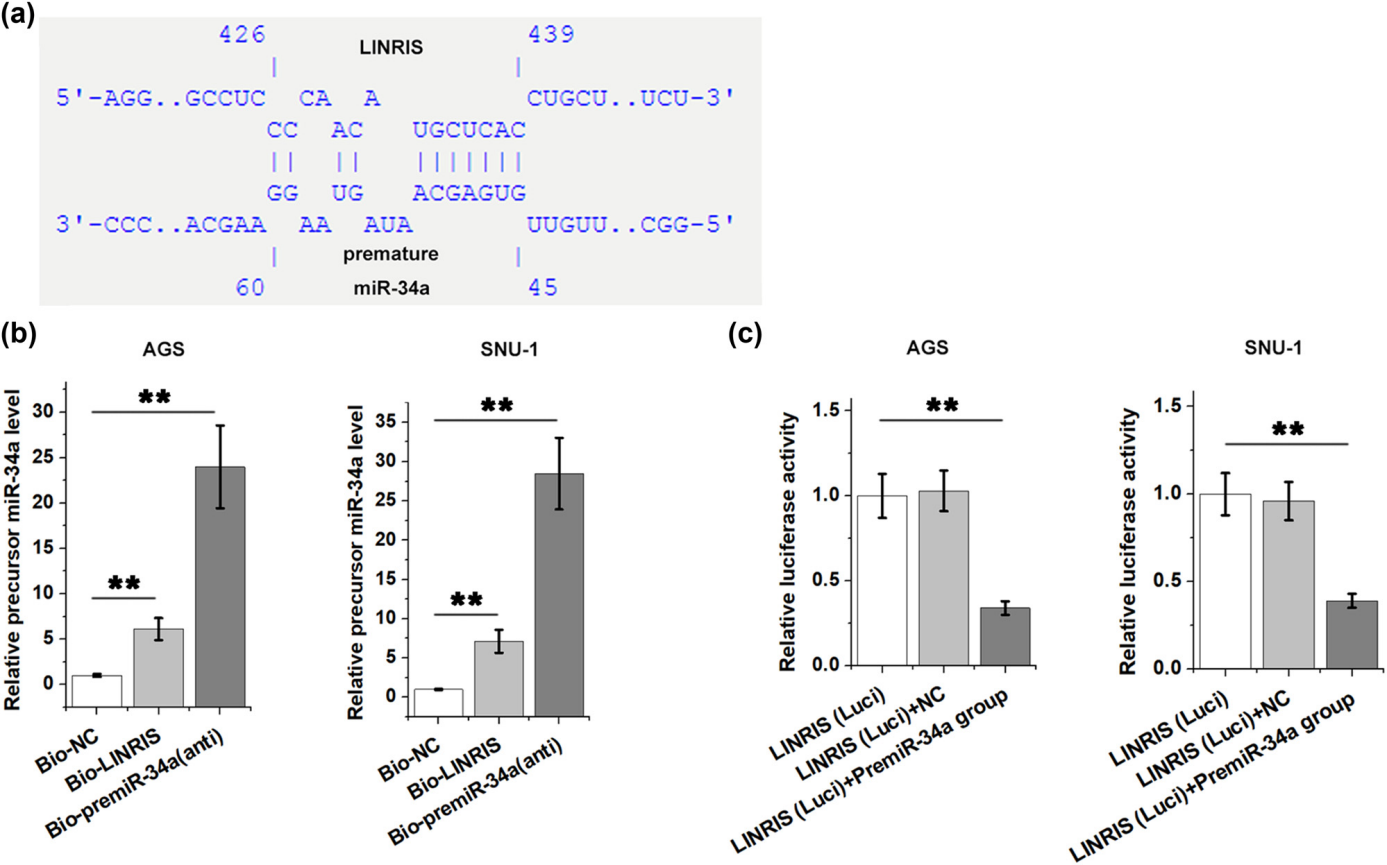
LINRIS could sponge precursor miR-34a in GC cells. The potential interaction between LINRIS and precursor miR-34a was predicted by IntaRNA 2.0 (a). RNA–RNA pull-down assay (b) and dual luciferase activity assay (c) were performed to further confirm the direct interaction between them (b). **p < 0.01.

Gene function is partially determined by subcellular location. To this end, the subcellular location of LINRIS in AGS cells was analyzed using nuclear fractionation assay. The data showed that LINRIS was expressed in both nuclear and cytoplasm samples (data not shown). The potential interaction between LINRIS and precursor miR-34a was predicted by IntaRNA 2.0, which revealed a strong potential interaction between them (Figure 5a). RNA–RNA pull-down assay was performed to further confirm their direct interaction. Compared to Bio-NC group, precursor miR-34a RNA level was significantly higher in Bio-LINRIS group and Bio-premiR-34a (anti) group (a positive control), confirming the direct interaction between LINRIS and precursor (Figure 5b, *p* < 0.01). Dual luciferase activity assay showed that precursor miR-34a (LINRIS (Luci) + PremiR-34a group), but not NC miRNA (LINRIS (Luci) + NC) reduced the luciferase activity produced by pGL2 Luciferase Reporter Vector containing the binding site of precursor miR-34a on LINRIS at the 5ʹ upstream of the luciferase gene (LINRIS (Luci)).

## 4 Discussion

lncRNA LINRIS is known to participate in CRC and NSCLC. LINRIS was upregulated in CRC and NSCLE tissues from patients. And the inhibition of LINRIS suppressed the proliferation of both tumors [13,14]. Zhu found that high LINRIS levels predicted poor survival of NSCLC patients and LINRIS silencing inhibited cell proliferation while miR-10a overexpression increased cell proliferation and inhibited the role of LINRIS silencing [14]. In the study, Wang et al. reported that LINRIS was overexpressed in CRC and could promote aerobic glycolysis by stabilizing IGF2BP2, thereby increasing cancer cell growth [13]. However, the expression and role of LINRIS in GC have not been reported.

MiR-34a plays a tumor-suppressive role in many cancers. MiR-34a is known to promote both *in vivo* and *in vitro* cell apoptosis by targeting certain oncogenes, such as SNAI1 [17,18]. It has been reported that miR-34a is downregulated in GC and has the potential to target survivin, thereby suppressing cancer cell proliferation [17]. The transcriptional activation of miR-34a plays a contributory role in apoptosis and glucose metabolism mediated by p53 [19,20]. However, there is no research on the relationship between miR-34a and LINRIS. In our study, we found that LINRIS was upregulated in GC. Further experiments showed that downregulation of LINRIS could positively regulate cancer cell apoptosis, suggesting the oncogenic role of LINRIS in GC. We also showed that LINRIS could negatively regulate mature miR-34a production but not miR-34a precursor in GC cells. In addition, the regulatory role of LINRIS in miR-34a maturation also affected GC cell apoptosis.

The negative correlation between lncRNA and mature miRNA and the irrelevance with precursor miRNA may be derived from their complex interactions and regulatory mechanisms. First, the negative correlation between lncRNA and mature miRNA may be due to their joint participation in the process of gene expression regulation. Both lncRNA and miRNA can bind to mRNA inside cells, affecting the stability, transcription, or translation process of mRNA. If lncRNA and miRNA bind to the same mRNA, they may compete with each other, resulting in the inhibition of one’s function. Therefore, when the expression level of lncRNA increases, it may reduce the binding of miRNA to mRNA, thus downregulating the regulatory effect of miRNA and *vice versa*. This competitive relationship may lead to a negative correlation between lncRNA and mature miRNA.

However, the reason why there is no direct correlation between lncRNA and precursor miRNA may lie in the significant differences in structure and function between precursor miRNA and mature miRNA. Precursor miRNA is an intermediate product in the process of miRNA generation, which needs to undergo a series of shearing and processing steps to become a functional mature miRNA. During this process, precursor miRNA does not possess the specific structure and function of mature miRNA, so they cannot interact directly with lncRNA or other RNA molecules like mature miRNA. In addition, the regulatory role of lncRNA may be more focused on the post-transcriptional level, affecting gene expression by interacting with mRNA, protein, or other RNA molecules. While precursor miRNA is mainly involved in the biogenesis of miRNA, its regulatory role may occur at the transcriptional level or an earlier stage. Therefore, there is a lack of direct interaction and regulatory relationship between lncRNA and precursor miRNA. This is the reason for the negative correlation between LINRIS and mature miR-34a, as well as the lack of correlation with precursor miR-34a. MiR-34a is encoded by the negative strand of human chromosome 1 [21]. UCSC Genome Browser (hg17 assembly) presentation of the location of pre-miR-34a on chromosome 1, the sequence of the miR-34a precursor (pre-miR-34a), located in the second exon. And the structural differences between the miR-34a precursor and the mature miR-34a are significant, leading to distinct biological functions.

Worth noting is that the direct interaction between LINRIS and precursor miR-34a was confirmed by both dual luciferase assay and RNA pulldown assay in our study. However, whether the miR-34a precursor plays a role in LINRIS-regulated apoptosis has not been further studied. Neither have we analyzed whether there is a correlation between GC staging and the expressions of LINRIS and miR-34. In addition, this study lacks *in vitro* research. Nevertheless, our research found that LINRIS suppresses the production of mature miR-34a while having no impact on the miR-34a precursor in GC cells. It has also been proved that the negative regulatory effect of LINRIS on mature miR-34a can regulate the apoptosis of GC cells. Furthermore, we have demonstrated the direct interaction between LINRIS and the precursor of miR-34a. These findings provide some guidance for peers, and the aforementioned unresolved issues are also worthy of further study.

## 5 Conclusion

This study demonstrates that LINRIS suppresses the production of mature miR-34a, while having no impact on the miR-34a precursor in GC cells. And there is a direct interaction between LINRIS and precursor miR-34a. Furthermore, LINRIS’ regulatory function in miR-34a maturation influences GC cell apoptosis.

## Abbreviation

GC: gastric cancer
